# ﻿Two new nematode species of the genus *Zalonema* (Desmodorida, Desmodoridae, Desmodorinae) from mangrove wetlands, China

**DOI:** 10.3897/zookeys.1260.164776

**Published:** 2025-11-20

**Authors:** Yuzhen Chen, Yuqing Guo

**Affiliations:** 1 Fisheries College, Jimei University, Xiamen 361021, China Jimei University Xiamen China; 2 Fujian Provincal Key Laboratory of Marine Fishery Resources and Eco-Environment, Jimei University, Xiamen 361021, China Jimei University Xiamen China

**Keywords:** Free-living marine nematode, Hainan Province, mangrove, meiofauna, new species, taxonomy, *Zalonema eurysbucca* sp. nov., *Zalonema cylindribucca* sp. nov.

## Abstract

Two new species of the genus *Zalonema* Cobb, 1920 were identified during a meiofaunal survey of mangrove sediment in China. *Zalonema eurysbucca***sp. nov.** is characterized by body length 1772–2309 µm, cuticle with transverse annuli; cephalic setae measuring 1–2 µm, and eight subcephalic setae (2–3 µm) arranged in a circle between the cephalic setae and the anterior margin of the amphid; amphideal fovea (4–4.5 turns) located laterally on the main posterior portion of the cephalic capsule; buccal cavity large and cup-shaped, with twelve cheilorhabdia in the vestibule, a cuticularized dorsal tooth, and minute ventral tooth; males possess lateral and ventral alae; spicule length (as arc) about 1.13–1.34 times the anal body diameter (abd); a small cup-shaped preanal supplement is present, and the ventral ala exhibit pore-like depressions at the somatic setae positions. *Zalonema cylindribucca***sp. nov.** is distinguished by the following characteristics: body length 1158–1451 μm; cuticle with transverse annuli; four cephalic setae (2–3 µm) and eight subcephalic setae (2–3 µm) arranged in separate circular rows; amphideal fovea with 3–3.5 turns; cylindrical buccal cavity containing 12 cheilorhabdia arranged circularly in the vestibule; a large dorsal tooth located near the cheilorhabdia base and a small ventral tooth at the buccal cavity’s base; males possess both lateral alae and ventral ala, spicules measuring 1.07–1.19 times the anal body diameter (as arc), a small cup-shaped preanal supplement present, and the ventral ala exhibit small pore-like depressions at somatic setae positions.

## ﻿Introduction

Meiofaunal sediments were collected during a preliminary survey of the meiofauna in mangrove mudflats adjacent to the cities of Danzhou and Sanya, Hainan Province, China. Nematodes are the dominant group at these two mangrove sampling stations, of which *Zalonema* Cobb,1920 is dominant genus, accounting for 5.5% and 26.9% of the abundance, respectively. Despite their ecological prevalence, the taxonomic knowledge of free-living marine nematodes in Chinese mangrove wetlands remains limited, with fewer than 50 species recorded to date (Chen et al. 2023; Zhu et al. 2023; Sun and Huang 2024).

The genus *Zalonema* Cobb,1920 belongs to the subfamily Desmodorinae within the family Desmodoridae. It was initially established as a family by Cobb based on the species *Zalonema nudum* Cobb, 1920. Gerlach (1963) considered *Zalonema* to be one of the subgenera of *Desmodora* and *Zalonema nudum* to be a synonym of *Desmodora megalosoma* Steiner, 1918. Later, Verschelde and Vincx (1996) raised *Zalonema* to genus level.

*Zalonema* is distinguished from other genera of the subfamily Desmodorinae by the following synapomorphies: (1) finely annulate cuticle lacking longitudinal striations or lateral differentiation; (2) well-developed, rounded triangular cephalic capsule; (3) large multispiral amphideal fovea (≥2 turns); and (4) presence of subcephalic setae (Tchesunov 2013; Verschelde and Vincx 1996; Verschelde et al. 1998). While the elongated body form and annulation pattern resemble those of representative genera of the subfamily Spiriniinae Chitwood, 1936, *Zalonema* is unequivocally differentiated by its distinct cephalic capsule, a structure entirely absent in Spiriniinae (Verschelde and Vincx 1996).

Recent taxonomic studies have revealed morphological variability within the genus, particularly in the position of subcephalic setae. The diagnosis of the genus by Verschelde and Vincx (1996) stated that the subcephalic setae are located in front of or at anterior edge of amphids, however seven recently described species, i.e.: *Zalonema iranicum* Gharahkhani, Pourjam, Leduc & Pedram, 2021, *Zalonema supplementorum* Gharahkhani, Pourjam, Leduc & Pedram, 2021, *Zalonema granda* Fadeeva, Mordukhovich & Zograf, 2016, *Zalonema kamchatkaensis* Fadeeva, Mordukhovich & Zograf, 2016, *Zalonema mariae* Larrazábal-Filho, Silva & Esteves, 2015, *Zalonema sesokoensis* Leduc, 2022 and *Zalonema vicentei* Larrazábal-Filho, Silva & Esteves, 2015, demonstrate subcephalic setae either aligned with, or posterior to, the amphids (Leduc 2022). Furthermore, Gharahkhani et al. (2021) proposed an intrageneric division of *Zalonema* into two groups based on the presence (Group I) or absence (Group II) of lateral or ventral alae in the posterior body region of males. Group I included species with lateral and/or ventral alae in the posterior body of males, group II species lack lateral or ventral alae in the posterior body region of males.

In this study, two new species of the genus *Zalonema* were found during a preliminary survey of the meiofauna in mangrove mudflats of China and are described.

## ﻿Material and methods

Sediment samples were collected from mangrove mudflats adjacent the cities of Danzhou (19.77–19.78°N, 109.26–109.27°E) and Sanya (18.23°N, 109.62°E), Hainan Province, China, and immediately preserved in 5% formaldehyde-seawater solution. Nematodes were extracted from the sediment by decantation and/or Ludox centrifugation in the laboratory, and stained with Rose Bengal for more than 24 h in the laboratory (Warwick et al. 1998). Sediment sampling and nematode extraction were performed as described in our previous studies (Chen et al. 2023).

Measurements are in µm. Abbreviations are as follows: *a* = body length/maximum body diameter; *b* = body length/pharynx length; *c* = body length/tail length; abd = anal body diameter; cbd = corresponding body diameter; vbd = vulval body diameter; *c*’ = tail length/abd; V = vulva from the anterior end; *V* (%) = position of vulva as % of body length from anterior end, that is, V/body length. All holotypes and paratypes are deposited in Jimei University, Xiamen, China.

## ﻿Results

**Order Desmodorida De Coninck, 1965**

**Family Desmodoridae Filipjev, 1922**

**Subfamily Desmodorinae Filipjev, 1922**

### ﻿Genus Zalonema

Taxon classificationAnimaliaDesmodoridaDesmodoridae

Cobb, 1920

18F6F558-07D2-52F5-8310-787658095DDD

#### Generic diagnosis.

(modified from Leduc 2022): Body long, cylindrical, gold-brown under light microscope with glass-like or golden cephalic capsule. Cuticular annuli close together, usually without ornamentation. Cephalic capsule rounded, triangular. Labial sensilla inconspicuous, 6+6, cephalic setae four, followed by four to eight subcephalic setae or papillae located anteriorly, posteriorly, or at same level as the amphids, amphideal fovea multispiral (two turns or more) located on the cephalic capsule. Short and small somatic setae arranged in several longitudinal rows. Pharynx cylindrical with pyriform basal bulb. Male reproductive system monorchic. Spicules slightly arched, cuticularized, without velum. Gubernaculum short, ventral and/or lateral alae sometimes present in males (ventral ala sometimes difficult to distinguish). Precloacal supplements present or absent. Female reproductive system didelphic, amphidelphic, with reflected ovaries. Tail conical. Caudal gland ducts and spinneret conspicuous.

### ﻿ Zalonema eurysbucca sp. nov.

Taxon classificationAnimaliaDesmodoridaDesmodoridae

7DF718D3-B5B7-5D54-8DE0-41783A2855D8

https://zoobank.org/054D7601-E14B-4EF1-BDB3-757BEDE2E58F

**Figure 1.**
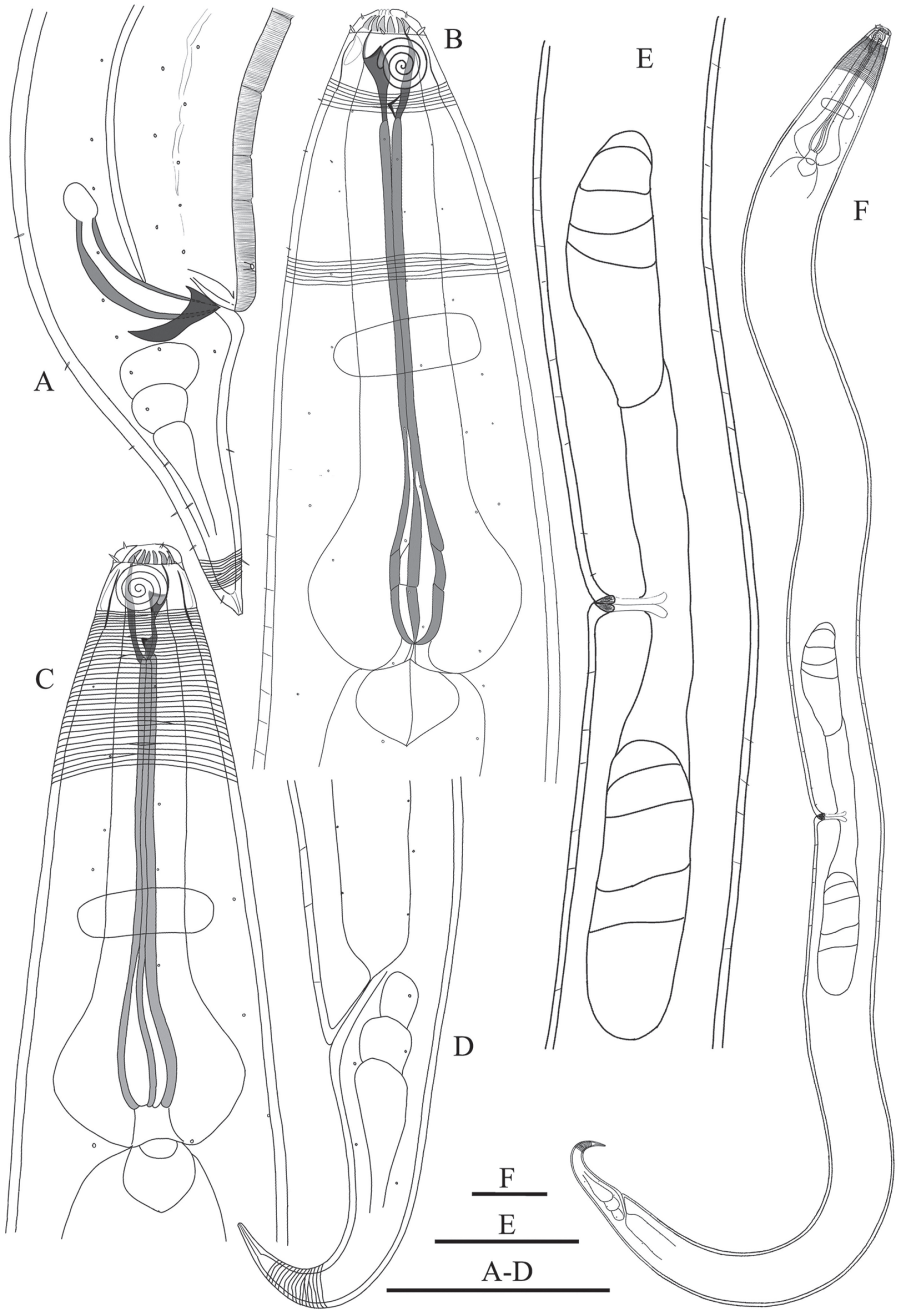
Drawing of *Zalonema eurysbucca* sp. nov. (lateral view). **A.** Male tail region, showing copulatory structure, ventral ala and preanal supplement; **B.** Male head end; **C.** Female head end; **D.** Female tail region; **E.** Female body part, showing ovaries; **F.** Female body. Scale bars: 50 μm (**A–D**); 125 μm (**E**); 100 μm (**F**).

**Figure 2.**
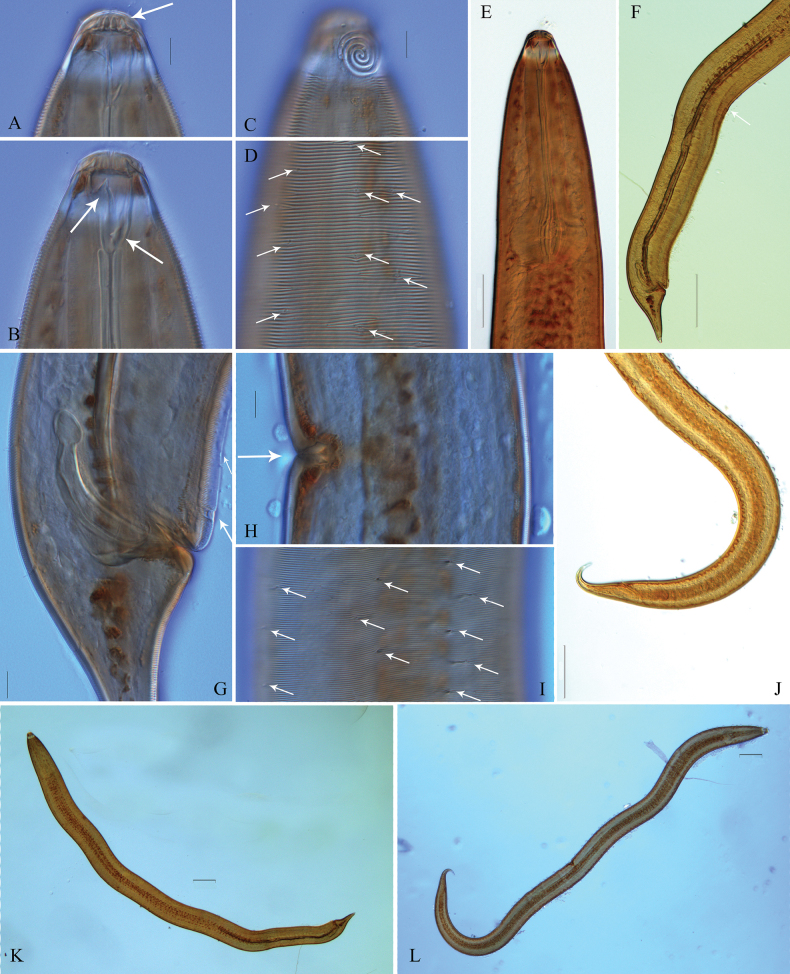
Microscopic images of *Zalonema eurysbucca* sp. nov. (lateral view). **A.** Male head region, showing cheilorhabdia in buccal cavity; **B.** Male head region, showing buccal cavity, large dorsal tooth and minute ventral tooth; **C.** Male amphid; **D.** Male anterior region, showing longitudinal rows of short somatic setae; **E.** Male pharynx and the pyriform posterior bulb; **F.** Male posterior region, showing lateral alae, the end of ventral ala and the tail; **G.** Male posterior region, showing copulatory structure, supplement and ventral ala; **H.** Female vulva; **I.** Female middle region, showing longitudinal rows of short somatic setae; **J.** Female tail; **K.** Male total body; **L.** Female total body. Scale bars: 10 μm (**A–D, G–I**); 50 μm (**E**); 100 μm (**F, J–L**)

Figs 1, 2; Tables 1, 2

#### Type material.

Six males and five females were collected from Station DZ in December, 2020. **Holotype**: • ♂1 on slide DZ20201214 2L18. **Paratypes**: • ♂2 on slide DZ20201214 2L101, • ♂3 and ♀1 on slide DZ20201214 2L111, • ♂4 on slide DZ20201214 2L102, • ♂5 on slide DZ20201214 1L113, • ♂6 on slide DZ20201214 2L117, • ♀2 and ♀3 on slide DZ20201214 2L18, • ♀4 on slide DZ20201214 2L110, • ♀5 on slide DZ20201214 1L101.

#### Type locality and habitat.

All specimens were collected from the muddy sediment in the mangrove reserve of Xinying Port in Danzhou City, Hainan Province, **China**. The main mangrove species here is *Bruguiera gymnorrhiza* (L.) Lam. Characteristics of surface sediments of sampling stations are shown in Table 1.

**Table 1.** Characteristics of surface sediments of sampling stations.

Station	Temperature (°C)	pH	Salinity (‰)
DZ20201214 1L	23.5	6.0	28
DZ20201214 2L	26	6.5	30

#### Etymology.

The species’ name is from the Latin (*Eurys* = broad) and refers to the large buccal cavity.

#### Measurements.

Morphological characteristics were observed and measured under a differential contrast microscopy (NIKON 80i) (Table 2).

**Table 2.** Individual measurements of *Zalonema eurysbucca* sp. nov. (in µm except for ratios).

Specimen	Holotype	Paratypes average (minimum-maximum)	
Characters	♂1	5 ♂♂	5 ♀♀
Body length	1926	2029 (1898–2309)	1969 (1772–2161)
Subcephalic setae cbd	23	26 (23–29)	23 (21–25)
Width of cephalic capsule	17	17 (16–19)	15 (13–17)
Length of cephalic setae	2	1.4 (1–2)	1.5 (2–2)
Length of subcephalic setae	3	2 (2–2)	2.5 (2–3)
Buccal cavity depth	34	38 (35–39)	37 (36–39)
Buccal cavity diameter	15	16 (13–19)	13 (12–15)
Amphids from anterior end	7	9 (7–10)	7 (6–8)
Amphid diameter	17	17 (15–18)	16 (15–17)
Amphid cbd	33	37 (30–41)	31 (27–37)
Amphid diameter/Amphid cbd (%)	52	46 (38–57)	52 (44–58)
Amphid turns	4.00	4.00 (4.00–4.00)	4.12 (4.00–4.50)
Nerve ring from anterior end	113	118 (106–134)	121 (110–132)
Nerve ring cbd	88	86 (75–96)	80 (73–88)
Pharynx length	224	218 (195–235)	205 (196–214)
Pharynx bulb diameter	76	76 (64–83)	66 (63–70)
Pharynx cbd	99	98 (83–107)	93 (88–98)
Max. body diameter	110	109 (94–122)	101 (91–108)
abd	64	59 (48–68)	43 (38–48)
Tail length	100	95 (87–99)	125 (116–136)
*c*’	1.57	1.61 (1.43–1.82)	2.95 (2.39–3.41)
Spicule length as chord	67	65 (56–69)	-
Spicule length as arc	76	73 (63–81)	-
Spicule length as arc/abd	1.19	1.25 (1.13–1.34)	-
Length of lateral alae	566	553 (505–611)	-
Length of gubernaculum	34	31 (28–34)	-
V	-	-	986 (903–1100)
vbd	-	-	94 (86–104)
*V* (%)	-	-	50 (48–51)
*a*	17.51	19.11 (16.19–20.63)	19.59 (16.41–21.96)
*b*	8.60	9.54 (9.30–10.64)	9.61 (8.31–10.87)
*c*	19.30	21.85 (21.07–23.65)	15.71 (14.55–16.67)

#### Description.

**General characteristics**: Golden-brown body elongated and cylindrical, with blunt and rounded anterior extremity and conical tail. Cuticle with distinct fine annuli arranged transversely from the posterior margin of the cephalic capsule to the tail, ending in a smooth, non-annulated tail tip approximately 10 µm in length. Somatic setae short, about 1–2 µm in length, arranged in eight longitudinal rows, and each seta is connected to an epidermal gland. Cephalic capsule consists of two parts: an anterior labial region, with a thinner cuticle; and a main posterior portion, with a conspicuously thickened cuticle without annulations, which is about 15–19 µm wide in males and about 13–16 µm wide in females. Inner labial sensilla not observed; six outer labial sensilla are papilliform, arranged in a circle; four cephalic setae arranged in a circle, about 1–2 µm in length, located slightly to the back of the external labial sensilla; eight subcephalic setae, about 2–3 µm in length, arranged in a circle between the cephalic setae and the anterior margin of the amphid. Large spiral amphideal fovea and aperture, 4–4.5 turns, located laterally on the main posterior portion of the cephalic capsule, with a diameter of about 38–58% of cbd. Buccal cavity large, with a maximum diameter of about 12–19 µm and a depth of about 34–39 µm. There are 12 cheilorhabdia arranged in a circle in the buccal cavity vestibule; the inner wall of the buccal cavity is sclerotized, with two teeth: one large dorsal tooth located at about one-third to half the depth of the buccal cavity, and one small ventral tooth at the bottom of the buccal cavity. Cylindrical pharynx with cuticularized lumen and pyriform posterior bulb; inner wall of the pharynx is thickened or sclerotized, and the pharyngeal bulb is broken into two parts. Nerve ring located at 50–63% of the length of the pharynx. Secretory-excretory system not observed.

**Males**: lateral alae present on both sides of posterior body part, extending from 505–566 µm in front of anus to a position on either side of the body at the anus. Ventral ala present, extending from 250–356 µm in front of anus to the anus. Reproductive system monorchic with single anterior outstretched testis located to the right of intestine. Spicules paired, equal, arcuate, length 1.13–1.34 times abd as arc, with proximal capitulum, without velum. Gubernaculum simple, without apophyses. A small cup-shaped preanal supplement located about 13 µm anterior to the anus. The surface of the ventral ala is marked by small pore-like depressions located at the positions corresponding to somatic setae. However, no distinct setal structures were observed within these depressions. These structures appear to correspond to the “small, pore-like, not cuticularized” features reported by Leduc (2022). In the present study, these pore-like depressions were clearly observed to be arranged regularly along the ventral ala, extending anteriorly along the body, and aligning with the pattern of somatic setae in regions where ventral ala are absent. In addition, each pore-like depression extends subcutaneously and connects to the epidermal gland, similar to the association seen in somatic setae. Therefore, we interpret these pore-like structures on the ventral ala not as preanal supplements, but likely as pore-like sensilla that may be homologous to somatic setae. Preanal setae not observed. Conical tail without terminal setae, length about 1.43–1.82 times abd. Spinneret with single opening present.

**Females**: Most characteristics are similar to those of the males, but the tail is longer than that of the males, with a length of about 2.4–3.4 times abd. Without lateral alae or ventral ala. Reproductive system didelphic, reflexed and present on the right side of the intestine. Vulva sclerotized, located at 48–51% of the body length.

#### Diagnosis.

*Zalonema eurysbucca* sp. nov. is distinguished by the following combination of characters: golden-brown body (length 1772–2309 µm) with a finely annulate cuticle; short somatic setae arranged in eight longitudinal rows; eight subcephalic setae (2–3 µm) arranged in a circle between the cephalic setae (1–2 µm) and the amphid’s anterior margin; amphideal fovea (4–4.5 turns) located laterally on the cephalic capsule’s posterior portion (diameter 38–58% of cbd); a large, cup-shaped buccal cavity with twelve cheilorhabdia, a cuticularized dorsal tooth, and a minute ventral tooth; and a conical tail. Males possess lateral and ventral alae; the arcuate spicules measure 1.13–1.34 times the anal body diameter (abd); gubernaculum simple without apophyses; a small cup-shaped preanal supplement, and the surface of the ventral ala at the position of the somatic setae sunken into small pore-like depressions.

In accordance with the classification proposed by Gharahkhani et al. (2021), the new species *Zalonema eurysbucca* sp. nov. belongs to Group I, which is characterized by the presence of lateral alae and/or ventral ala on the posterior part of the male body. *Zalonema eurysbucca* sp. nov. is distinguished by the combined presence of both lateral and ventral alae, contrasting with *Zalonema ditlevseni* (Micoletzky, 1922) Gerlach, 1963 and *Zalonema megalosoma* (Steiner, 1918) Gerlach, 1963, which possess only lateral alae and lack a ventral ala (in contrast to Gharahkani’s classification that places *Zalonema megalosoma* in Group II, the redescription of this species by Gerlach (1963) reports the presence of lateral alae in males); and from *Zalonema myrianae* Verschelde & Vincx, 1996, which lacks lateral alae but possesses ventral ala. *Zalonema eurysbucca* sp. nov. can also be differentiated from other species in Group I. *Zalonema eurysbucca* sp. nov. differs from *Zalonema mariae* Larrazábal-Filho, Silva & Esteves, 2015 in having eight subcephalic setae (vs four), a cup-shaped buccal cavity with a single large dorsal (at 30–50% depth) and small ventral teeth (vs. elongate buccal cavity with two dorsal teeth, one after the other), and spicules lacking a velum (Larrazábal-Filho et al. 2015); from *Zalonema vicentei* Larrazábal-Filho, Silva & Esteves, 2015 in the subcephalic setae position (between cephalic setae and the anterior margin of the amphid vs. at amphid posterior margin) and number (8 vs. 4) (Larrazábal-Filho et al. 2015); from *Zalonema iranicum* Gharahkhani, Pourjam, Leduc & Pedram, 2021 in the dental formula (one dorsal and one ventral tooth vs 3 teeth, a large anterior dorsal tooth, a small posterior dorsal tooth, and a medium-sized ventral-subventral tooth) (Gharahkhani et al. 2021); from *Zalonema supplementorum* Gharahkhani, Pourjam, Leduc & Pedram, 2021 in the subcephalic setae count (8 vs 4) and amphid coils (4–4.5 vs 3 turns) (Gharahkhani et al. 2021); and from *Zalonema sesokoensis* Leduc, 2022 by lacking amphid sexual dimorphism and possessing a larger buccal cavity (Leduc 2022).

### ﻿ Zalonema cylindribucca sp. nov.

Taxon classificationAnimaliaDesmodoridaDesmodoridae

319735FB-4F40-53BA-910C-3EC3D17B4286

https://zoobank.org/D9F11052-B88B-4498-AA92-82C0BBF8BA33

Figs 3, 4; Table 3

#### Type material.

Five males and three females were collected from Station SY in July, 2020. **Holotype**: • ♂1 on slide SY2020730 2H108. **Paratypes**: • ♂2 & ♀1 on slide SY2020730 1L126, • ♂3 & ♀2 on slide SY2020730 2H102, • ♂4, • ♂5 & ♀3 on slide SY2020730 2H108.

#### Type locality and habitat.

All specimens were collected from the muddy sediment in the mangrove reserve of Yalong Bay Qingmei Port in Sanya City, Hainan Province, China. The primary mangrove species here are *Rhizophora stylosa* Griff. and *Avicennia marina* (Forssk.) Vierh.

#### Etymology.

The species is name for its cylindrical buccal cavity.

#### Measurements.

Morphological characteristics were observed and measured under a differential contrast microscopy (NIKON 80i) (Table 3).

**Table 3.** Individual measurements of *Zalonema cylindribucca* sp. nov. (in µm except for ratios).

Specimen	Holotype	Paratypes average (minimum-maximum)	
Characters	♂1	4 ♂♂	3 ♀♀
Body length	1451	1299 (1231–1364)	1291 (1158–1371)
Subcephalic setae cbd	21	20 (19–21)	20 (19–21)
Width of cephalic capsule	10	9.5 (9–10)	9 (8–10)
Length of cephalic setae	3	2.3 (2–3)	2 (2–2)
Length of subcephalic setae	3	2.5 (2–3)	2.7 (2–3)
Buccal cavity depth	29	29 (28–31)	29 (28–30)
Buccal cavity diameter	6	5.3 (5–6)	5.3 (5–6)
Amphids from anterior end	9	8.8 (8–10)	8.3 (7–9)
Amphid diameter	9	8.5 (8–9)	8.3 (8–9)
Amphid cbd	25	23 (21–24)	23 (22–24)
Amphid diameter/Amphid cbd (%)	37	38 (35–41)	37 (36–38)
Amphid turns	3.00	3.50 (3.50–3.50)	3.33 (3.25–3.50)
Nerve ring from anterior end	111	105 (101–109)	100 (96–102)
Nerve ring cbd	62	56 (54–59)	58 (51–64)
Pharynx length	178	169 (163–174)	165 (161–168)
Pharynx bulb diameter	47	46 (42–50)	46 (44–49)
Pharynx cbd	65	62 (59–66)	63 (59–67)
Max body diameter	69	67 (64–70)	72 (67–78)
abd	42	39 (36–44)	31 (29–34)
Tail length	84	79 (74–85)	105 (98–111)
*c*’	2.02	2.07 (1.76–2.32)	3.39 (3.25–3.65)
Spicule length as arc	55	53 (49–56)	-
Spicule length as chord	46	44 (41–47)	-
Spicule length as arc/abd	1.11	1.14 (1.07–1.19)	-
Length of gubernaculum	20	22 (21–23)	-
Length of lateral alae	410	385 (374–391)	-
Distance of supplement from anus	9	9 (8–10)	-
V	-	-	693 (626–732)
vbd	-	-	69 (66–73)
*V* (%)	-	-	54 (53–54)
*a*	21.09	19.36 (17.89–20.11)	17.96 (17.33–18.92)
*b*	8.17	7.73 (7.19–8.09)	7.81 (7.21–8.18)
*c*	17.17	16.48 (15.88–17.17)	12.27 (10.81–13.69)

#### Description.

**General characteristics**: Body elongate-cylindrical, golden-brown in color, with a cephalic capsule and a conical tail. Cuticle with annuli arranged transversely from the posterior margin of the cephalic capsule to the tail, with tail tip about 10 µm smooth and without annuli. The annuli are spaced by about 0.6 µm at both the anterior and posterior ends of the body, and by about 0.8 µm in the middle part. Somatic setae short, 1–2 µm in length, and arranged in eight longitudinal rows. Cephalic capsule divided into two parts: an anterior labial region, with a thinner cuticle; and a main posterior portion, with a width of 8–10 µm, and its cuticle distinctly thickened without annuli. No inner labial sensilla were observed; the six outer labial sensilla are papilliform and arranged in a circle; four cephalic setae and eight subcephalic setae are each arranged in a circle, about 2–3 µm in length, with the cephalic setae located slightly posterior to the outer labial sensilla; the subcephalic setae are positioned between the cephalic setae and the anterior margin of the amphid. Large spiral amphideal fovea and aperture, 3–3.5 turns, located laterally on the main posterior portion of the cephalic capsule, with diameter about 35–41% of cbd. Buccal cavity cylindrical, with a maximum diameter of approximately 5–6 µm and a depth of about 28–31 µm. There are 12 cheilorhabdia arranged in a circle in the buccal cavity vestibule. At the anterior part of the buccal cavity, near the base of the cheilorhabdia, there is a large dorsal tooth; and another small ventral tooth is located at the bottom of the buccal cavity, close to the esophagus. Cylindrical pharynx with cuticularized lumen and a pear-shaped posterior bulb; the inner wall of the pharynx is thickened or sclerotized, and the pharyngeal bulb slightly broken. Nerve ring located at 60–65% of the pharynx. The excretory system was not observed.

**Figure 3.**
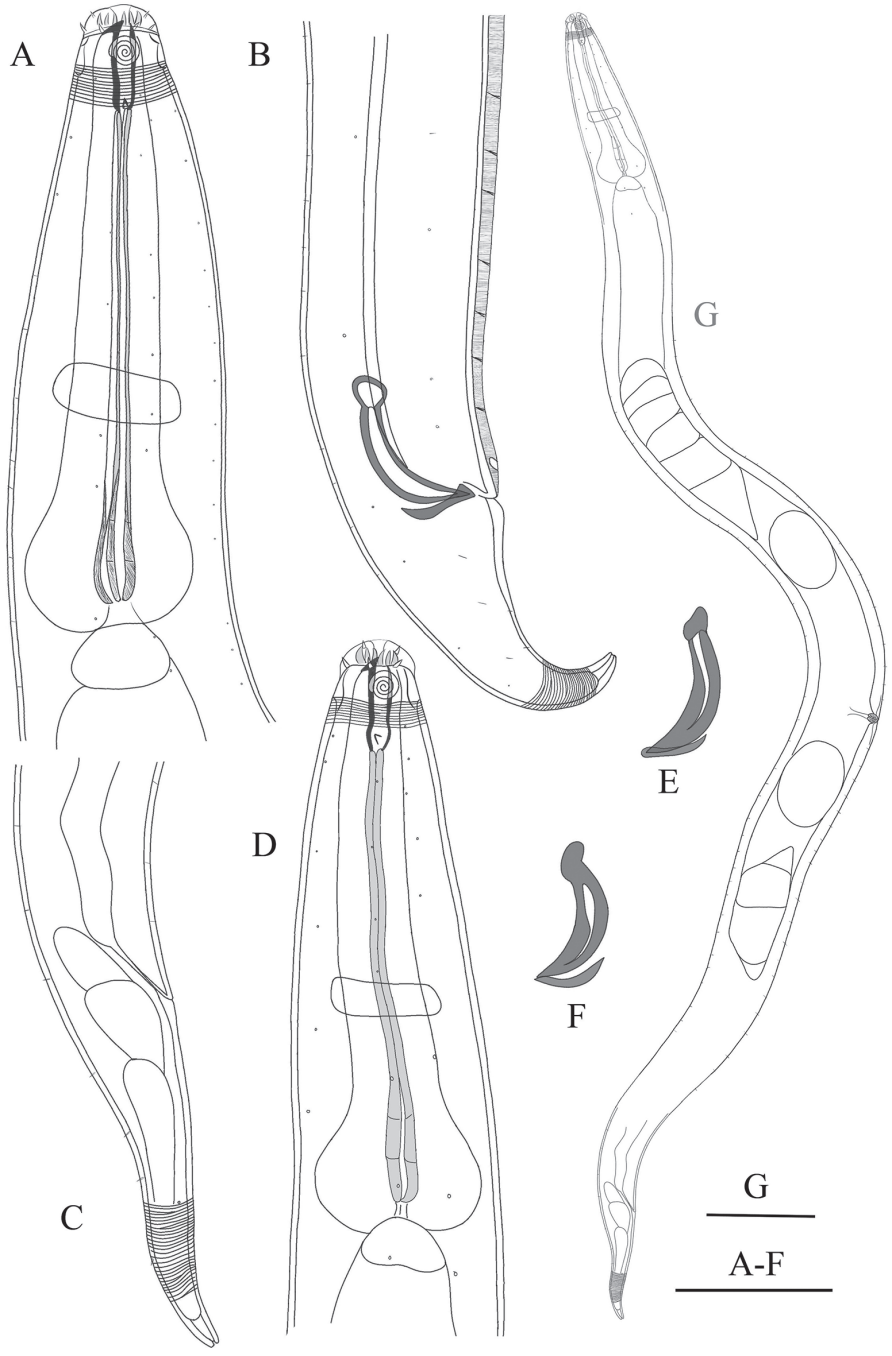
Drawing of *Zalonema cylindribucca* sp. nov. (lateral view). **A.** Male head end; **B.** Male tail region, showing copulatory structure, ventral ala and preanal supplement; **C.** Female tail region; **D.** Female head end; **E, F.** Male copulatory structure; **G.** Female body; Scale bars: 50 μm (**A–F**); 100 μm (**G**).

**Males**: lateral alae present on both sides of posterior body part, extending from 374–410 µm in front of anus to a position on either side of the body at the anus. Ventral ala present, extending from 290–420 µm in front of anus to the anus. Reproductive system monorchic with single anterior outstretched testis located to the right of intestine. Spicules paired, equal, arcuate, length about 1.07–1.19 times abd as arc, with proximal capitulum. Gubernaculum simple, without apophyses. A small cup-shaped preanal supplement located about 8–10 µm anterior to the anus. The surface of the ventral ala is marked by small pore-like depressions located at the positions corresponding to somatic setae. However, no distinct setal structures were observed within these depressions. Preanal setae not observed. Conical tail without terminal setae, length about 1.76–2.32 times abd. Spinneret with single opening present.

**Figure 4.**
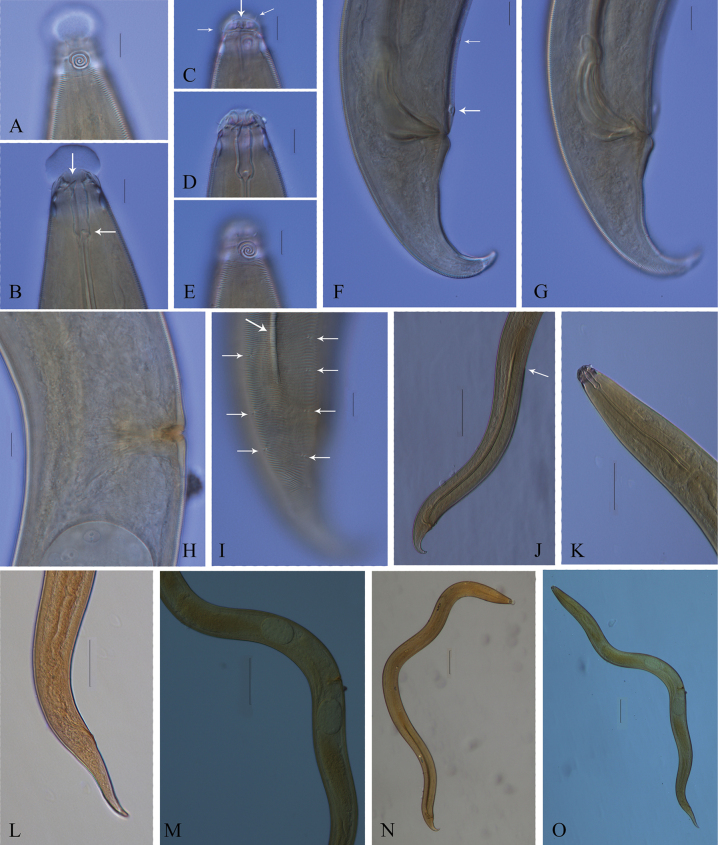
Microscopic images of *Zalonema cylindribucca* sp. nov. (lateral view). **A.** Male amphid; **B.** Male buccal cavity and teeth; **C.** Female head region, showing cheilorhabdia in buccal cavity and cephalic setae, subcephalic setae; **D.** Female head region, showing buccal cavity, and teeth; **E.** Female amphid; **F.** Male tail region, showing tail, supplement and ventral ala; **G.** Male copulatory structure; **H.** Female vulva; **I.** Male posterior region, showing lateral alae and longitudinal rows of short somatic setae; **J.** Male posterior region, showing lateral alae, the end of ventral ala and the tail; **K.** Female anterior region, showing pharynx and the posterior bulb; **L.** Female tail; **M.** Female ovaries; **N.** Male total body; **O.** Female total body. Scale bars: 10 μm (**A–I**); 50 μm (**K, L**); 100 μm (**J, M–O**).

**Females**: Most characteristics are similar to those of the males, but the tail is longer than that of the males, with a length of about 3.25–3.65 times abd. Without lateral alae or ventral ala. Reproductive system didelphic, reflexed and present on the right side of the intestine. Vulva sclerotized, located at 53–54% of the body length.

#### Diagnosis.

*Zalonema cylindribucca* sp. nov. is distinguished by its golden-brown body color, length 1158–1451 μm; cuticle with annuli; somatic setae short and arranged in eight longitudinal rows; four cephalic setae and eight subcephalic setae are each arranged in a circle, about 2–3 µm in length; amphid spiral and 3–3.5 turns, with diameter about 35–41% of cbd; buccal cavity cylindrical, 12 cheilorhabdia arranged in a circle in the buccal cavity vestibule; a large dorsal near the base of the cheilorhabdia, and another small ventral tooth at the bottom of the buccal cavity; male with both lateral alae and ventral ala, spicules length about 1.07–1.19 times abd as arc, gubernaculum without apophyses, a small cup-shaped preanal supplement present, and the surface of the ventral ala at the position of the somatic setae sunken into small pore-like depressions.

According to the classification by Gharahkhani et al. (2021), *Zalonema cylindribucca* sp. nov. belongs to Group I, which is characterized by having lateral alae and/or a ventral ala on the posterior part of the male body. The new species has both lateral alae and ventral ala on the posterior part of the male body. Therefore, it can be distinguished from *Zalonema ditlevseni* (Micoletzky, 1922) Gerlach, 1963 and *Zalonema megalosoma* (Steiner, 1918) Gerlach, 1963 (which only have lateral alae but no ventral ala) and *Zalonema myrianae* Verschelde & Vincx, 1996 (which lacks lateral alae but has a ventral ala). In addition, compared with other species in Group I, the new species can be differentiated based on the number and position of subcephalic setae, the shape of the buccal cavity and the number and position of teeth in the buccal cavity, the number of turns of the amphid, and the morphology of the spicules (Larrazábal-Filho et al. 2015; Gharahkhani et al. 2021; Leduc 2022).

*Zalonema cylindribucca* sp. nov. and *Zalonema eurysbucca* sp. nov. are quite similar in terms of the presence of preanal supplement, spicules, and the existence of lateral alae and ventral ala. However, the two species differ significantly in the shape of the buccal cavity and the location of the large dorsal tooth. *Zalonema eurysbucca* sp. nov. has a large buccal cavity that is wider at the top and narrower at the bottom, with the large dorsal tooth located at a depth of about one-third to one-half of the oral cavity. In contrast, *Zalonema cylindribucca* sp. nov. has a cylindrical buccal cavity that is almost equal in width at the top and bottom, with the large dorsal tooth situated at the anterior part of the buccal cavity, near the base of the cheilorhabdia. Additionally, the body size and relative size of the amphid differ between the two species, *Zalonema eurysbucca* sp. nov. has a body length of 1772–2309 μm, with a relatively shorter tail (male c = 19.3–23.65, female c = 14.55–16.67). *Zalonema cylindribucca* sp. nov. has a body length of 1158–1451 μm, with a relatively longer tail (male c = 15.88–17.17, female c = 10.81–13.69).

## ﻿Discussion

There are 15 species have been described in this genus, including the newly described species in the present study. However, *Zalonema nudum* Cobb, 1920 has been synonymized with *Zalonema megalosoma* (Steiner, 1918) Gerlach, 1963 (Verschelde et al. 1998), while *Zalonema propinqua* (Allgén, 1951) Gerlach, 1963 remains a *species inquirendum* (Verschelde et al. 1998; Larrazábal-Filho et al. 2015; Fadeeva et al. 2016; Leduc 2022). Since Fadeeva et al.’s (2016) key to eight species, three additional taxa have been described: two from the Persian Gulf (Gharahkhani et al. 2021), one from Sesoko Island, Japan (Leduc 2022). In addition, two new species from mangrove mudflats in China are described in the present study. Consequently, the genus currently comprises 13 valid species. All valid species are listed below.

### ﻿List of valid *Zalonema* species

*Zalonema ditlevseni* (Micoletzky, 1922) Gerlach, 1963

*Zalonema granda* Fadeeva, Mordukhovich & Zograf, 2016

*Zalonema iranicum* Gharahkhani, Pourjam, Leduc & Pedram, 2021

*Zalonema kamchatkaensis* Fadeeva, Mordukhovich & Zograf, 2016

*Zalonema maldivensis* (Gerlach, 1963) Verschelde et al., 1998

*Zalonema mariae* Larrazábal-Filho, Da Silva & Esteves, 2015

*Zalonema megalosoma* (Steiner, 1918) Gerlach, 1963

*Zalonema myrianae* Verschelde & Vincx, 1996

*Zalonema sesokoensis* Leduc, 2022

*Zalonema supplementorum* Gharahkhani, Pourjam, Leduc & Pedram, 2021

*Zalonema vicentei* Larrazábal-Filho, Da Silva & Esteves, 2015

*Zalonema eurysbucca* sp. nov.

*Zalonema cylindribucca* sp. nov.

The dichotomous key to species of the genus *Zalonema* was revised here based on Fadeeva et al (2016) and Gerlach (1963), which considers male morphological characters only. The key incorporates all currently valid species, including the newly described taxa (Gerlach 1963, 1964; Verschelde et al. 1998; Larrazábal-Filho et al. 2015; Fadeeva et al. 2016; Gharahkhani et al. 2021; Leduc 2022). Complementary to the key, Table 4 summarizes their diagnostic characteristics.

**Table 4.** Differentiating characters of all known *Zalonema* species.

**Species**	*Zalonema ditlevseni*		*Zalonema granda*		*Zalonema iranicum*		*Zalonema kamchatkaensis*		*Zalonema maldivensis*	
**Characteristic**										
Specimen sexual	♂	♀	♂	♀	♂	♀	♂	♀	♂	♀
N	1	13	10	3	9	8	4	2	1	1
L(μm)	1590	1570	3310–4277	3310–3932	1940–3000	2130–3152	1956–2778	2271–2610	2175	1513
*a*	42	41	55–91	46–105	27.7–35.6	28.2–40.4	34.0–42.0	31.5–34.9	47	31
*b*	7.6	10.5	7.6–11.9	7.8–12.0	8.3–12.6	9.5–11.0	8.0–11.1	9.7–10.0	9.6	8.1
*c*	15.1	16.4	14.5–31.5	13.8–24.6	16.6–22.6	15.9–20.5	18.0–21.0	18.0–21.0	13	16.3
CS(μm)	5	-	6–8	6–7	2.0–3.0	2.0–3.0	4	4	7	
Sub CS(μm)	-	-	6–8	6–8	-	-	3–5	3–4	6	
Ad(μm)	7	-	17–21	15–19	14.0–15.0	13.0–16.0	30–45	37–43	7	
Pl(μm)	210	-	302–371	332–349	222–253	217–285	245–293	245–268	225	186
A%	37	25	40–50	40–43	50.0–62.5	50.0–61.5	55–69	36–43	25–33	
At	2.5–3	-	2.2–2.3		4.0–4.5	4.0–5.0	2.1–2.2		2.5	
Sc/abd	1.3*	-	1.4–1.6	-	1.1–1.4	-	1.3–1.8	-	1.2–1.4	-
Sc(μm)	46	-	77–84	-	54–64	-	64–79	-	40–45	-
Gl(μm)	24	-	27–42	-	21–28	-	15–25	-	1/2 Sc	-
abd(μm)	35	-	43–58	45–54	54–64	33–39	45–51	36–44	39	28
*c*’	3	-	2.4–3.2	2.6–3.4	2.1–2.9	3.2–4.5	2.3–3.4	2.7–3.8	2.4–3.3	
Tl(μm)	105	-	134–162	134–155	110–135	115–158	115–142	123–137	90*	82*
Sn	0	-	0	-	0	-	0	-	10–11	-
*V* (%)	-	51	-	64.0–65.0	-	47.0–50.8	-	57.0–62.0	-	50
la	presence	-	absence	-	presence	-	absence	-	absence	-
va	absence	-	absence	-	presence	-	absence	-	absence	-
Reference	(Gerlach 1964)	(Micoletzky 1922)	(Fadeeva et al. 2016)		(Gharahkhani et al. 2021)		(Fadeeva et al. 2016)		(Gerlach 1963)	
**Species**	*Zalonema mariae*		*Zalonema megalosoma*		*Zalonema myrianae*		*Zalonema sesokoensis*			
**Characteristic**										
Specimen sexual	♂	♀	♂	♀	♂	♀	♂	♀		
N	19	25	2	1	2	4	1	2		
L(μm)	1080–2640	936–2580	1482;1938	1541	1583	1402–1589	2040	1396;1110		
*a*	22.5–35	19–28	34;24	24	36.8;37.7	29.2–35.9	39	29;26		
*b*	6–9.5	6–10	9.3;9	8.6	9.6;9.8	8.4–10.2	12	8		
*c*	15–25	11–15	12.1;19	9.3	15.7;15.1	11.8–14	21	11;12		
CS(μm)	5–9	4–7	4–6.5	4–6.5	4;3	4–5	7	5–6		
Sub CS(μm)	3–5.5	3.5–7	5–6	5–6	-	-	4–5	3–7		
Ad(μm)	-	-	13–16	9.5	7	6–7	13	8;7		
Pl(μm)	141–331.5	133–279	160;220	180	164;161	156–167	167	177;132		
A%	32–61	22–46	55–60	50	38;35	32–39	50	36;39		
At	3.5	2.5	3	3	2	2 or more	3.5	2.5		
Sc/abd	-	-	1.1–1.3	-	1.0–1.1	-	1.67*	-		
Sc(μm)	39–75	-	47–66	-	38;43	-	70	-		
Gl(μm)	18–43.5	-	30	-	16;20	-	20	-		
abd(μm)	34.5–82.5	48–118	35;65	33	34;41	28–30	42	27;24		
*c*’	1.5–3	2–4	1.6–3.5	5	3.0;2.6*	-	2.4	4.7;3.9		
Tl(μm)	60–100.5	69–151	122;58*	165*	101;105	110–126	99	127;94		
Sn	0	-	1	-	0	-	7	-		
*V* (%)	-	48–69	-	45	-	46–49	-	48		
la	presence	-	presence #	-	absence	-	presence	-		
va	presence	-	absence #	-	presence	-	presence	-		
Reference	(Larrazábal-Filho et al. 2015)		(Gerlach 1963)		(Verschelde and Vincx 1996)		(Leduc 2022)			
**Species**	*Zalonema supplementorum*		*Zalonema vicentei*		*Zalonema eurysbucca* **sp. nov.**		*Zalonema cylindribucca* **sp. nov.**			
**Characteristic**										
Specimen sexual	♂	♀	♂	♀	♂	♀	♂	♀		
N	8	7	10	10	6	5	5	3		
L(μm)	1254–1605	1337–1685	1065–2175	1245–2130	1898–2309	1772–2161	1231–1451	1158–1371		
*a*	20.6–30.4	17.5–22.5	21.5–36	21–29	16.19–20.63	16.41–21.96	17.89–21.09	17.33–18.92		
*b*	8.0–10.0	8.4–9.2	7–10	9.5–11.5	8.6–10.64	8.31–10.87	7.19–8.17	7.21–8.18		
*c*	16.7–21.5	13.0–14.4	15–22.5	10–16	19.3–23.65	14.55–16.67	15.88–17.17	10.81–13.69		
CS(μm)	1.5–3.0	2.0–3.0	4–7	4–6	1–2	1–2	2–3	2–3		
Sub CS(μm)	1.0–2.0	1.0–2.0	3–7	3.5–6	2–3	2–3	2–3	2–3		
Ad(μm)	6.0–7.5	6.0–7.5	-	-	15–18	15–17	8–9	8–9		
Pl(μm)	149–165	163–197	135–223.5	132–210	195–235	196–214	163–178	161–168		
A%	27.3–36.8	27.3–37.5	30–57	29–60	38–57	44–58	35–41	36–38		
At	3.0–3.2	2.8–3.2	3	3	4	4–4.5	3–3.5	3.25–3.5		
Sc/abd	1.2–1.3	-	-	-	1.13–1.34	-	1.07–1.19	-		
Sc(μm)	41–43	-	37.5–79.5	-	63–81	-	41–47	-		
Gl(μm)	15–17	-	15–33	-	28–34	-	20–23	-		
abd(μm)	32–34	25–32	34.5–63	28–55	48–68	38–48	36–44	29–34		
*c*’	2.2–2.7	3.3–4.7	1.5–3	2.5–4	1.43–1.82	2.39–3.41	1.76–2.32	3.25–3.65		
Tl(μm)	70–87	93–120	57–102	111–147	87–100	116–136	74–85	98–111		
Sn	12–16	-	0	-	1	-	1	-		
*V* (%)	-	51.1–55.7	-	37–60	-	48–51	-	53–54		
la	presence	-	presence	-	presence	-	presence	-		
va	presence	-	presence	-	presence	-	presence	-		
Reference	(Gharahkhani et al. 2021)		(Larrazábal-Filho et al. 2015)		This study		This study			

Notes: *: data calculated from formula; -: data absent; #: According to the redescription of *Zalonema megalosoma* by Gerlach (1963), males of this species possess lateral alae, which is inconsistent with Gharahkani’s subdivision of the genus. While ventral ala was not mentioned, but the preanal cuticle was reported to be ventrally thickened; *N*: number of specimens; L: body length; CS: length of cephalic setae; Sub CS: length of subcephalic setae; Ad: amphid diameter; Pl: pharynx length; A%: amphid diameter/amphid cbd (%); At: amphid turns; Sc: spicule length as arc; Gl: length of gubernaculum; Tl: tail length; Sn: number of supplement; la: lateral alae; va: ventral ala.

### ﻿Key to species of the genus *Zalonema*

**Table:** 

1	Male with lateral alae and/or ventral ala on posterior part	**2**
–	Male without lateral alae or ventral ala on posterior part	**11**
2	Males with either lateral alae or ventral ala	**3**
–	Males with both lateral alae and ventral ala	**5**
3	Male with only lateral alae	**4**
–	Male with only ventral ala	***Zalonema myrianae***
4	Amphids 0.5–0.6 of head width in size. Pharyngeal bulb onion-shaped, wider than long	***Zalonema megalosoma***
–	Amphids 0.25–0.4 of head width in size. Pharyngeal bulb less onion-shaped. Tail with two ventral groups of short setae	***Zalonema ditlevseni***
5	Sexual dimorphism of fovea amphidialis present	**6**
–	Sexual dimorphism of fovea amphidialis absent	**7**
6	Four subcephalic setae located at posterior margin of amphid; buccal cavity with two dorsal teeth, but without ventral tooth; male without supplement	***Zalonema mariae***
–	Eight subcephalic setae located anterior of amphid; buccal cavity with ventral tooth; male with seven precloacal supplements, posteriormost supplement cup-shaped, cuticularized and conspicuous, other supplements small, pore-like	***Zalonema sesokoensis***
7	Buccal cavity with three teeth	**8**
–	Buccal cavity with two teeth (one dorsal and one ventral)	**9**
8	Male with 12–16 pore-like precloacal supplements; amphidial fovea spi­ral with 3.0–3.2 turns	***Zalonema supplementorum***
–	Male without supplement; amphidial fovea spiral with 4–4.5 turns	***Zalonema iranicum***
9	Buccal cavity with one small dorsal tooth and one small ventral tooth; four subcephalic setae	***Zalonema vicentei***
–	Buccal cavity with one large dorsal tooth and one small ventral tooth; eight subcephalic setae	**10**
10	Buccal cavity wider at the top than the bottom, with one large dorsal tooth and one small ventral tooth. Large dorsal tooth is located approximately one-third to half the depth of buccal cavity	***Zalonema eurysbucca* sp. nov.**
–	Buccal cavity cylindrical, with one large dorsal tooth and one small ventral tooth. Large dorsal tooth is located in front of buccal cavity, near base of cheilorhabdia	***Zalonema cylindribucca* sp. nov.**
11	Male with supplement	***Zalonema maldivensis***
–	Male without supplement	**12**
12	Length of the body 2–2.7 mm, male amphideal fovea 50% of cbd, subcephalic setae papillose	***Zalonema kamchatkaensis***
–	Length of the body 3.3–4.3 mm, male amphideal fovea 50% of cbd or less, subcephalic setae long	***Zalonema granda***

## Supplementary Material

XML Treatment for Zalonema

XML Treatment for Zalonema eurysbucca

XML Treatment for Zalonema cylindribucca

## ﻿Additional information

We would like to thank the anonymous reviewers for their valuable comments on the manuscript and our teammate Mingming Zou for her assistance in providing specimens.

### Use of AI

No use of AI was reported.

### Author ORCIDs

Yuzhen Chen https://orcid.org/0009-0009-8953-0756

Yuqing Guo https://orcid.org/0000-0002-9177-0144

### Data availability

All of the data that support the findings of this study are available in the main text.
